# Barriers and facilitators for the uptake of the national INOCA recommendation among Dutch cardiologists: A survey study

**DOI:** 10.1016/j.ijcrp.2026.200718

**Published:** 2026-10-08

**Authors:** Irene Göttgens, Lianne C.G. Roelofs, Jojanneke Smeenk, Linda Modderkolk, Sabine Oertelt-Prigione

**Affiliations:** a Department of Primary and Community Care, Radboud Institute for Medical Innovation, Radboud University Medical Center, Nijmegen, the Netherlands; b AG 10 Sex- and Gender-Sensitive Medicine, Medical Faculty OWL, University of Bielefeld, Bielefeld, Germany

**Keywords:** INOCA, Guideline implementation, Cardiology, Gender differences

## Abstract

**Background:**

In 2020, the Dutch Society for Cardiologists (NVVC) published a national recommendation for diagnostics and treatment of Ischaemia with Non-Obstructive Coronary Arteries (INOCA), a historically underrecognized condition. In this study we identify determinants of implementation of the NVVC INOCA recommendation through a national survey among Dutch cardiologists.

**Methods:**

We performed a cross-sectional, nationwide online survey among Dutch cardiologists, incorporating attitudinal statements derived from prior qualitative research and the Measurement Instrument for Determinants of Innovation (MIDI) questionnaire. Descriptive analyses were used to identify facilitators and barriers to implementation. Multiple regression analyses examined associations between attitudinal factors and familiarity with the recommendation.

**Results:**

A total of 134 cardiologists participated in this study with 78 (58%) completing the long version of the questionnaire and 56 (42%) the short version. Facilitators for implementation were predominantly user-related, driven by positive outcome expectations, perceptions of professional responsibility, and perceived communicative capabilities. In contrast, key barriers were primarily organizational, including the absence of institutional policies, implementation coordination, and performance feedback. Familiarity with the recommendation was independently associated with perceived improvements in INOCA-related knowledge, confidence in communicating under clinical uncertainty, and gender.

**Conclusions:**

Uptake of the NVVC INOCA recommendation among Dutch cardiologists is largely motivated by individual professional commitment and positive outcome expectations but is constrained by limited organizational support. Strengthening institutional implementation structures and reinforcing professional communicative capabilities may help translate high individual motivation into consistent peer-dissemination and recommendation-concordant INOCA care.

## Highlights

• 42% of cardiologists are unaware of the INOCA recommendation despite treating patients.
• Facilitators driven by individual motivation; barriers are predominantly organizational.
• Women cardiologists report higher awareness and familiarity with the recommendation.
• Gender predicts familiarity with the recommendation, alongside knowledge and communicative confidence.

## 1 Introduction

Ischaemia with non-obstructive coronary arteries (INOCA) is increasingly recognized as a clinically relevant and burdensome condition. Patients with INOCA present with anginal symptoms but lack flow-limiting coronary stenosis on angiography. Once considered benign, accumulating evidence demonstrates that INOCA is associated with impaired quality of life, recurring hospitalizations and adverse cardiovascular outcomes [[1], [2], [3]]. Recent patient surveys further reveal substantial symptom burden and reduced quality of life, particularly among women, highlighting both clinical and gender-related disparities [4,5]. Despite these findings, the diagnosis and management of INOCA remain challenging. Both coronary microvascular dysfunction and vasospasm require specialised testing, yet these procedures are not consistently applied or available in routine care [6,7].

Reflecting these challenges, the Dutch Society of Cardiology (NVVC) issued one of the first national INOCA management recommendations in 2020 to support cardiologists in recognizing, diagnosing, and treating this patient population [8]. However, the publication of a recommendation does not necessarily ensure implementation. Uptake of clinical recommendations and guidelines in healthcare are shaped by characteristics related to ease-of-use, professional attitudes and institutional- and peer support among others [9,10].

Our previous qualitative study among Dutch cardiologists identified a range of determinants influencing the uptake of the INOCA recommendation, including both structural and attitudinal factors [11]. These ranged from structural issues, such as diagnostic availability, to epistemic and cultural dimensions, such as professional recognition of INOCA as a legitimate clinical condition. The present study extends this work by quantifying the prevalence of the eight previously identified attitudinal factors (four facilitating and four hampering) across the broader Dutch cardiovascular workforce through a national survey.

This study has three aims: 1) to evaluate key determinants for NVVC INOCA recommendation implementation among Dutch cardiologists; 2) to quantify the prevalence of eight previously identified attitudinal factors; and 3) to examine their association with recommendation familiarity.

## 2 Methods

### 2.1 Study design and setting

We conducted a national, cross-sectional online survey study among cardiologists in the Netherlands. This study followed up on our prior qualitative research exploring cardiologists’ perceptions of the NVVC INOCA recommendation uptake. We now tested those findings with a larger sample of cardiologists and incorporated a broader evaluation of determinants for effective implementation of the INOCA recommendation.

### 2.2 Participants and recruitment

All cardiologists practicing in the Netherlands (approximately 1100) were eligible to participate [12]. A multimodal recruitment strategy was employed. Invitations were distributed via the professional networks of cardiologists, through mailing lists and institutional channels. Participation was voluntary and anonymous. The survey was open between April and September 2025. Respondents provided informed consent digitally before accessing the online questionnaire. A full description of our recruitment strategy is included in the supplementary material.

### 2.3 Survey instrument

The questionnaire consisted of 59 items covering three sections: 1) professional background, 2) attitudinal statements that facilitate and hamper the uptake of the INOCA recommendation, and 3) implementation determinants (MIDI questionnaire) [9,11].

Respondents unfamiliar with the INOCA recommendations or not treating INOCA patients completed a shortened version containing only background items (section 1) as the NVVC INOCA recommendation was not considered used or implemented in these cases. The full survey is provided in the supplementary material.

Attitudinal items were derived from the eight factors (four facilitating, four hampering) identified in our prior qualitative study [11]. The factors were transformed into attitudinal statements and scored on a 5-point Likert scale (disagree/agree). The facilitating factors included: 1) perceived improvement in knowledge and structure of the diagnostic and treatment process for people with INOCA, 2) acknowledgement and legitimization of patient's symptoms and complaints, 3) ability to communicate effectively in the midst of clinical uncertainty, 4) advocacy and support of colleagues and experts in INOCA patient care. Barrier factors included: 1) Perceived insufficiency of knowledge about INOCA, 2) Perceived lack of effective diagnostic and treatment options for people with INOCA, 3) Stereotyping of INOCA patients as ‘typical’ women, 4) Caring for INOCA patients is perceived as demanding and complicated.

Broader implementation determinants were assessed using the Measurement Instrument for Determinants of Innovation (MIDI) [9]. The MIDI is an instrument designed to identify determinants that facilitate or hinder the implementation of innovations (such as practice recommendations and guidelines) in healthcare practices. The survey evaluates determinants across four domains: 1) Innovation domain (e.g. procedural clarity, level of complexity, congruence with current practice), 2) User domain (e.g. outcome expectations, task perception, satisfaction), 3) Organizational domain (e.g. available time and resources, feedback structures and organizational embedding), and 4) Socio-political environment (e.g. legislation and regulations). Respondents rated items on a 5-point Likert scale.

#### 2.3.1 Survey branching

Cardiologist who reported both (1) awareness of the availability of the INOCA recommendation and (2) regular care for INOCA patients were directed to the full survey (all three sections). Cardiologists who indicated either no awareness of the INOCA recommendation and/or not treating patients with INOCA in practice received a short survey consisting of demographics items only.

This design ensured that MIDI items were only distributed among cardiologists with direct exposure to both the recommendation and patients with INOCA in clinical practice.

### 2.4 Data collection

The survey was administered online between (April – September 2025). Participants provided informed consent prior to participation. Average completion time was estimated at 10 min for the full version and 2-3 min for the short version. Data were collected anonymously and processed in accordance with the General Data Protection Regulation (GDPR).

### 2.5 Data analysis

Analysis was performed using SPSS (version 29). Descriptive statistics summarized respondent characteristics and survey responses. Attitudinal items and MIDI determinants were descriptively analyzed for respondents who completed the full survey. In line with previous studies that used the MIDI survey, positive responses (totally agree or agree) were classified as facilitator when ≥80% of cardiologists (totally) agreed [[13], [14], [15], [16], [17], [18]]. Negative responses (totally disagree or disagree) were classified as barrier when ≥ 20% of cardiologists (totally) disagreed. Multiple regression analysis examined which attitudinal facilitator predicted the level of familiarity with the INOCA Recommendation. Familiarity was operationalized using the single questionnaire item: “*To what extent are you familiar with the content of the NVVC INOCA guidelines and the specific recommendations contained therein?“*, which are scored on a 5-point Likert scale ranging from “*I know the guidelines and recommendations by heart and can apply them without consulting the text.”* To “*I am not very familiar with the guidelines and always have to consult them when applying the recommendations*”. P values < 0.05 were considered statistically significant.

## 3 Results

### 3.1 Participant characteristics

A total of 134 cardiologists (46 women and 88 men) completed our survey, yielding an estimated response rate of 12%. A subtotal of 56 (42%) cardiologists (12 women (21.4%), 44 men (78.6%)) completed the short version of the questionnaire; 54 (96%) of them did not know or had not read the recommendation, nevertheless 49 (87,5%) were treating patients with (suspected) INOCA.

A subtotal of 78 (58%) cardiologists (34 women (43.6%), 44 men (56.4%)), who were aware of the INOCA recommendation and treated INOCA patients in their clinic, completed the full version of the survey. Most cardiologists had between 6 and 15 years of experience in clinical practice and mainly worked in a peripheral hospital setting. Overall, women cardiologists reported more awareness of the availability of INOCA recommendation (93% compared to 77%) and read them more thoroughly, compared to men cardiologists (52% compared to 24%) (Table 1). A respondent flowchart is included in the supplementary material.

**Table 1:** Participant characteristics.

N (%)	Total (N = 134)	Short version (n = 56)	Full version (n = 78)	Women cardiologists (n = 46)	Men cardiologists (n = 88)
**Gender**					
Woman	46 (34.3%)	12 (21.4%)	34 (43.6%)		
Man	88 (65.7%)	44 (78.6%)	44 (56.4%)		
**Clinical experience** (in years)					
< 1	4 (3.0%)	2 (3.6%)	2 (2.6%)	1 (2.2%)	3 (3.4%)
1-5	22 (16.4%)	8 (14.3%)	14 (17.9%)	9 (19.6%)	13 (14.8%)
6-15	60 (44.8%)	23 (41.1%)	37 (47.4%)	26 (56.5%)	34 (38.6%)
>15	48 (35.8%)	23 (41.1%)	25 (32.1%)	10 (21.7%)	38 (43.2%)
**Type of clinical cardiology care setting**					
Peripheral hospital	86 (64.2%)	38 (67.9%)	48 (61.5%)	27 (58.7%)	59 (67.0%)
University hospital	29 (21.6%)	13 (23.2%)	16 (20.5%)	10 (21.7%)	19 (21.6%)
Independent treatment center	18 (13.4%)	4 (7.1%)	14 (17.9%)	9 (19.6%)	9 (10.2%)
Other	1 (0.7%)	1 (1.8%)	-	-	1 (0.7%)
**Extent of awareness of the content of the NVVC INOCA Recommendation**					
I am not aware of the recommendations.	23 (17.2%)	23 (41.1%)	0	3 (6.5%)	20 (22.7%)
I am aware of the recommendation but have not read it.	31 (23.1%)	31 (55.4%)	0	8 (17.4%)	23 (26.1%)
I am aware of the recommendation and have read it superficially.	35 (26.1%)	0	35 (44.9%)	11 (23.9%)	24 (27.3%)
I am aware of the recommendation and have read it thoroughly and in full.	45 (33.6%)	2^a^ (3.6%)	43 (55.1%)	24 (52.2%)	21 (23.9%)
**Treating patients with a (suspected) INOCA diagnosis**					
Yes	127 (94.8%)	49 (87.5%)	78 (100%)	43 (93.5%)	84 (95.5%)
No	7 (5.2%)	7 (12.5%)	0	3 (6.5%)	4 (4.5%)

a Two cardiologists indicated that they were familiar with the guidelines but did not treat patients with INOCA in their daily clinical practice. Therefore, they completed the shortened questionnaire.

### 3.2 Determinants for implementation of the NVVC INOCA recommendation (MIDI questionnaire)

Facilitators for the implementation of the NVVC INOCA Recommendation in clinical practice were located most prominently in the user domain (i.e. in cardiologists themselves) and related to outcome expectations. Most of the cardiologists reported that the recommendation supports adequate recognition (98.7%), diagnosis (89.7%) and treatment (98.7%) of patients with (suspected) INOCA. Most cardiologists also indicated that using the recommendation is part of their professional responsibility (89.7%) and that they possess sufficient knowledge to apply it in daily clinical practice (91%). Notably, women cardiologists perceived the recommendation as easier to use and considered the recommendation more relevant for their patients with (suspected) INOCA compared to men cardiologists (Table 2).

**Table 2:** Perceived facilitators to implementation of the NVVC INOCA Recommendation (MIDI survey).

Domain	Determinant	Item	Women (%)	Men (%)	Total (%)
User	Outcome expectations	Expect adequate recognition of INOCA	**97.1**	**100**	**98.7**
User	Outcome expectations	Expect adequate treatment of INOCA	**100**	**97.7**	**98.7**
User	Outcome expectations	Expect adequate diagnosis of INOCA	**91.2**	**88.6**	**89.7**
User	Professional obligation	Responsibility to use guideline	**91.2**	**88.6**	**89.7**
Innovation	Complexity	Guideline is easy to use^a^	**100**	77.3	**87.2**
User	Knowledge	Know enough to use guideline	**94.1**	**88.6**	**91.0**
Innovation	Relevance for client	Relevant for INOCA patients	**85.3**	72.7	78.2

a Reverse coded. Items with a scores of ≥ 80% agreement are categorised as facilitators.

Barriers to implementation of the NVVC INOCA Recommendation were most frequently reported in relation to the organizational context. A substantial proportion of the cardiologists indicated the absence of institutional policies or formal instructions for using the recommendation (74.4%), as well as a lack of arrangements for replacing experienced staff members when they leave the organisation (42.3%). In addition, most cardiologists reported that no designated person is responsible for the implementation of the INOCA recommendation within their organisation (65.4%), that concurrent organisational changes negatively affect the implementation of the recommendation (29.5%), and feedback about the uptake of the INOCA recommendation is not provided on a regular basis (57.7%).

Barriers were also identified among user-related determinants, particularly those associated with descriptive norms. Over one-third of the cardiologists (34.6%) perceived that none or a small minority of their colleagues for whom the recommendation is intended uses it. Furthermore, women cardiologists reported a modest barrier related to self-efficacy in implementing all the steps of the recommendation into practice and a relatively lower sensitivity to peer and leadership opinions when deciding to follow the INOCA recommendation. Men cardiologists instead reported a modest barrier in the perceived inadequate availability of personnel to implement the recommendation (Table 3). An overview of all determinants for implementation (facilitators, barriers and neutral MIDI items) is presented in the supplementary material.

**Table 3:** Perceived barriers to implementation of the NVVC INOCA Recommendation (MIDI survey).

Domain	Determinant	Item	Women (%)	Men (%)	Total (%)
Organisation	Formal ratification	No institutional policy	**73.5**	**75.0**	**74.4**
Organisation	Coordinator	No implementation coordinator	**70.6**	**61.4**	**65.4**
Organisation	Performance feedback	No feedback on uptake	**64.7**	**52.3**	**57.7**
Organisation	Replacement of staff	No timely replacement	**38.2**	**45.5**	**42.3**
User	Descriptive norms	Colleagues do not use the guideline	**38.2**	**31.8**	**34.6**
Organisation	Time available	Insufficient time	**26.5**	18.2	**21.8**
Organisation	Staff capacity	Enough staff capacity to use guideline	11.8	**22.7**	17.9
User	Subjective norms – motivation to comply	Supervisors' opinion to comply	**20.6**	13.6	16.7
User	Subjective norms – motivation to comply	Other professionals' opinion to comply	**20.6**	6.8	12.8
User	Self-efficacy	Unable to follow all steps	**20.6**	18.2	19.2
Innovation	Observability	Outcome not observable	**23.5**	11.4	16.7

Items with a score of ≤ 20% disagreement are categorised as barriers.

### 3.3 Attitudinal factors: prevalence of prior qualitative findings

Overall, responses to the INOCA statements reflected a largely positive attitude towards INOCA as a legitimate and clinically relevant condition. Most cardiologists reported that knowledge and treatment options have improved (92.3%) and almost all endorsed the importance of recognizing symptoms and taking patients’ complaints seriously (97.4%), with no significant differences between women and men cardiologists. The majority disagreed that the notion that insufficient knowledge undermines the legitimacy of INOCA as a clinical entity (93.6%). Cardiologists also reported a high confidence in their ability to communicate effectively with patients with INOCA in the midst of clinical uncertainty (94.9%). Perceptions regarding the availability of diagnostic tools and treatments options were more heterogeneous, with lower overall agreement. Most participants agreed that the perception of INOCA as primarily a ‘women's disease’ reflects stereotyping (78.2%), a view that was consistent across genders. With respect to professional support, 64.1% of cardiologists reported feeling supported by colleagues and INOCA experts, although lower mean scores were reported among women cardiologists (58.8%) compared to men cardiologists (68.2%). A substantial proportion of the participants perceived care of patients with INOCA as demanding and complex, with higher mean scores among women (59.8%) compared to men cardiologists (50%). Lastly, 37.2% of the cardiologist agreed that the current diagnostic tools and treatment options are insufficient (Table 4).

**Table 4:** Attitudinal statements regarding INOCA, stratified by gender.

Statement	Agreement (%) Total	Agreement women (%)	Agreement men (%)
I think it is important to recognize the symptoms and complaints and take them seriously. (F)	97.4	97.1	97.7
I notice that knowledge of the diagnosis and treatment process has improved in recent years. (F)	92.3	94.1	90.9
I am capable to communicate effectively with patients with (s)INOCA, even in de midst of clinical uncertainty. (F)	94.9	97.1	93.2
I think that not enough is known about INOCA, which makes me sceptical about whether it is a legitimate condition. (B)	6.4	5.9	6.8
I consider the perception that INOCA is primarily a women's disease to be a stereotype. (B)	78.2	76.5	79.5
I feel supported by colleagues and experts in the care I provide to people with (s)INOCA. (F)	64.1	58.8	68.2
I perceive caring for patients with (s)INOCA demanding and complicated. (B)	53.8	59.8	50.0
There are insufficient diagnostic tools and treatment options. (B)	37.2	35.3	38.6

Responses were measured on a 5-point Likert scale (1 = totally disagree; 5 = totally agree). Agreement reflects the proportion selecting “agree” or “strongly agree.” F = facilitator, B = barrier.

### 3.4 Associations between attitudinal factors and familiarity with the INOCA recommendation

In the multivariate model, adjusted for gender, years of experience, and type of clinical cardiology care setting, two attitudinal factors were independently associated with familiarity with the INOCA recommendation. Perceived improvement in knowledge of the diagnostic and treatment process was positively associated with familiarity (B = 0.54, 95% CI 0.24–0.84), as was the perceived ability to communicate effectively with patient with (suspected) INOCA (B = 0.47, 95% CI 0.14–0.80). Gender was also independently associated with familiarity, with men cardiologists reporting lower familiarity than women cardiologists (B = −0.47, 95% CI −0.86 to −0.09). The final model explained a moderate proportion of variance in familiarity with the recommendation (R^2^ = 0.318, adjusted R^2^ = 0.270) (Table 5).

**Table 5:** Association between attitudinal factors and familiarity with the NVVC INOCA recommendation.

Predictor	(unstandardized) B	95%CI
(Constant)	−0.42	−2.48; 1.64
I notice that knowledge of the diagnosis and treatment process has improved in recent years.	**0.54**	**0.24; 0.84**
I am able to communicate effectively with patients with INOCA, even in the midst of clinical uncertainty.	**0.47**	**0.14; 0.80**
Gender	**−0.47**	**−0.86; −0.09**
Years working as cardiologist	0.14	−0.11; 0.39
Type of healthcare institution employed	0.13	−0.11; 0.37

R^2^ = 0.318; Adjusted R^2^ = 0.270. Residuals approximately normally distributed; homoscedasticity acceptable; VIF <1.5 (indicating minimal multicollinearity). B represents the change in familiarity score per one-point increase on the 5-point Likert scale of the attitudinal item.

## 4 Discussion

### 4.1 Key findings

This national survey identifies a disconnect between clinical exposure and engagement with the INOCA recommendation, indeed, 42% of respondents were unaware of or had not read the NVVC INOCA recommendation, despite 87.5% of this group actively treating patients with suspected INOCA. This suggests that clinical exposure alone does not ensure engagement with available national guidance. Women cardiologists reported higher awareness (93% vs. 77%) and were more likely to have read the recommendation thoroughly (52% vs. 24%) than men cardiologists. Gender remained independently associated with familiarity with the recommendation in the multivariable analysis, indicating that the differences persist beyond variation in clinical experience and practice setting.

Implementation determinants, which were assessed among a subsample of cardiologists who were both familiar with the recommendation and treating patients, showed a clear distribution across domains. Facilitators were primarily located at the level of the individual clinician and the innovation itself, including positive outcome expectations, professional responsibility, perceived knowledge, and low complexity of the recommendation. Most cardiologists believed the recommendation supports adequate recognition, diagnosis, and treatment of patients with suspected INOCA. In contrast, barriers were predominantly organizational, including the absence of institutional policies, implementation coordinators, and regular feedback on uptake. A substantial proportion of cardiologists also perceived limited use of the recommendation among colleagues, suggesting that implementation has not been fully embedded within routine cardiology practice. This perception is supported by our key finding that 42% of the respondents were unaware or had not read the recommendations, while 87,5% of this subsample reported to be treating patients with (suspected) INOCA.

The attitudinal findings quantitatively verified themes identified in our preceding qualitative study. In that study, cardiologists described several attitudinal factors that, based on their clinical experiences, could either facilitate or hamper the uptake of the INOCA recommendation. The quantitative findings show that the facilitating attitudes were widely endorsed: most cardiologists considered INOCA a legitimate and clinically relevant condition, perceived improvements in diagnostic knowledge and treatment options, and reported confidence in communicating with patients despite clinical uncertainty. In contrast, potentially hampering attitudes identified in the qualitative study, such as skepticism regarding the legitimacy of INOCA or the perceived complexity of INOCA care, appeared less prevalent in the present cohort. Interpretation of these findings requires caution, however, as attitudinal factors were only assessed among cardiologists already aware of the recommendation and actively involved in INOCA care. This likely reflects a subgroup with greater pre-existing engagement and interest in INOCA, which may partly explain the relatively low prevalence of negative attitudinal barriers.

Not all attitudinal factors were equally relevant for implementation. Only perceived improvements in diagnostic and treatment knowledge and confidence in communicating with patients under clinical uncertainty independently predicted familiarity with the recommendation. Together, these findings suggest that uptake of the NVVC INOCA recommendation is currently driven predominantly by individual clinician motivation and perceived capability, while organizational integration and support remain limited.

### 4.2 Implementation context and clinical practice variation

Substantial variation in INOCA management has been reported internationally, with inconsistent access to specialised diagnostics and limited standardisation of care pathways [19]. Our findings align with prior work showing that lack of specialist INOCA training, experience with coronary function testing (CFT), and variations and inconsistency in institutional infrastructures constrains the implementation of emerging INOCA care guidance, even when professional motivation is high [[20], [21], [22]]. Barriers such as limited availability of diagnostic options, absence of local protocols, and insufficient reimbursement of microvascular testing continue to shape INOCA care in clinical practice. Although initiatives such as the Dutch NL-CFT registry represent important progress towards improving the INOCA knowledge base, access to advanced diagnostics remains uneven and institution-dependent [11,23].

Within established implementation frameworks, the findings reflect a familiar imbalance in the literature: motivation and capability at the individual clinician level appear relatively strong, whereas organisational/structural opportunities remain limited [24]. This pattern mirrors prior research showing that professional endorsement alone is insufficient to achieve sustained implementation without formalised institutional support. This is particularly relevant for clinicians who are already familiar with INOCA care, as in the population included in this study.

The greater engagement among women cardiologists with the NVVC INOCA recommendations in our study may reflect several complementary mechanisms. Some studies suggest that women physicians show slightly higher adherence to clinical guidelines compared to their male peers [25,26]. In addition, broader workforce dynamics may also contribute, whereby increasing representation of women cardiologists contributes to greater attention to historically under-recognized female-prevalent conditions such as INOCA [27]. Importantly, the gender imbalance in engagement with the recommendation in our study was more pronounced among those who were unaware or had not read the recommendation, whereas it decreased among those who were already familiar with the recommendation. This suggests that the observed gender differences may be relevant primarily at the early engagement stage, rather than representing major resistance to its content.

The timing of the NVVC INOCA recommendation may further explain this pattern. The recommendation was published in 2020, alongside the EAPCI Expert Consensus Document on Ischaemia with Non-Obstructive Coronary Arteries, which introduced European-level guidance on INOCA diagnosis and management [28]. It is plausible that men cardiologists may have engaged preferentially with the European consensus document over the national recommendation, while women cardiologists may have been more likely to engage with both channels. The subsequent publication of the 2024 European Society of Cardiology (ESC) Guidelines for the Management of Chronic Coronary Syndromes, which elevated invasive coronary function testing in INOCA patients to a Class I recommendation, may represent a validation threshold that triggers broader engagement regardless of gender [29]. Our survey, conducted approximately one year after this guideline publication, likely captures a transitional phase in which early adopters had already engaged with the earlier consensus documents, while broader awareness within the cardiology community may still have been evolving.

### 4.3 Implication for improving the uptake of the NVVC INOCA recommendations

The findings of this study suggest that improving the uptake of the NVVC INOCA recommendation among Dutch cardiologists will require strategies that address both awareness and organisational integration of INOCA care beyond passive dissemination. The observation that many cardiologists encounter patients with (suspected) INOCA but remain unfamiliar with the recommendation (42%), despite the availability of an e-learning module and digital materials accompanying the recommendation [8,30], indicated that dissemination of the national guideline has not yet fully reached clinicians involved in routine patient care. Importantly, as the MIDI survey was only administered among cardiologists who were both familiar with the recommendation and treated patient with (suspected) INOCA, the barriers and facilitators are reflecting the perspective of cardiologists already engaged with the recommendation. The prevalence of barriers among the broader workforce is likely higher than reported here.

While dedicated educational efforts might support uptake of the recommendation, they are unlikely to resolve the implementation gap. Greater improvement in uptake likely depends on strengthening organisational support for INOCA care and integrating it into existing practices. Indeed, uptake of clinical practice guidelines depends on a combination of continued medical education and multidisciplinary training, structural implementation through the development of INOCA-focused clinical pathways, cultural interventions and accountability [31]. Hospitals and cardiology departments could facilitate implementation by integrating the recommendation into local care pathways, clinical protocols, and quality improvement initiatives. The appointment of local implementation champions, providing support and performance feedback, and facilitating access to diagnostic modalities, i.e. through referral networks, may help increase provision of care compliant with the recommendation [32,33]. Cardiologists who treat patients with suspected INOCA and are familiar with the recommendation could act as ambassadors in the initial dissemination of INOCA expertise within their department and professional networks until critical knowledge diffusion is reached. Given the observed gender differences in early engagement with the guideline, peer-dissemination networks may play an important role in supporting a gender-balanced uptake across the workforce [34].

At the systemic level, establishment of designated INOCA expertise centers could serve as regional hubs for coordinated implementation, integrating national and international guideline evidence into locally adaptable frameworks and providing targeted continuing medical education to surrounding institutions. Improved alignment of reimbursement structures and the development of networks of specialised INOCA diagnostics may further support equitable access to appropriate care, which is especially relevant for women, who represent the majority of affected patients [21,35]. Together, such interventions would help improve implementation of the NVVC INOCA recommendation, moving from reliance on individual clinician motivation and initiative, towards a more structurally supported model of INOCA care.

### 4.4 Methodological considerations

This study builds on the findings of our prior qualitative work and applied a validated evaluation framework for determinants affecting implementation to the NVVC INOCA recommendation [9], allowing for a theory-informed assessment of recommendation uptake. Nevertheless, several limitations and methodological considerations can be discussed.

First, the response rate of approximately 12% (134 of 1100 registered Dutch cardiologists) limits generalizability to the broader cardiology workforce. While we conducted an extensive recruitment campaign through multiple professional and networking channels (supplementary materials), we could not determine the exact reach of the study invitations. Voluntary participation likely resulted in overrepresentation of cardiologists with greater interest in INOCA, and critically, those least engaged with the recommendation were least likely to participate. The prevalence of barriers identified in this study, including lack of familiarity with the recommendation (42%), is likely an underestimate of the true gap. Findings should therefore be interpreted as reflecting a relatively engaged subsample, and implementation challenges across the full workforce, particularly on the individual-level, may be more pronounced than reported here.

Second, determinants were classified as barriers or facilitators using the 80%/20% thresholds established in prior MIDI literature [[13], [14], [15], [16], [17], [18]]. While these thresholds reflect consensus conventions, classification is inherently sensitive at the boundary: determinants scoring just above or below a threshold (e.g., Time available: 21.8%; Self-efficacy: 19.2%) may shift classification with minor variation in individual responses. Such near-threshold items should therefore be interpreted as marginally classified rather than definitively categorised, and readers should exercise appropriate caution when drawing strong inferences from these specific determinants.

Third, familiarity with the recommendation was assessed using a single self-reported item. While multi-item scales are generally preferred for enhanced reliability, single-item measures are methodologically acceptable when the construct is concrete, unambiguous, and behaviourally anchored; as was the case in this study. Additionally, minimizing respondent burden was a deliberate design choice when recruiting healthcare practitioners. Nevertheless, future research may benefit from validated multi-item scales for greater measurement robustness.

Fourth, while this participant-to-predictor ratio of our regression model meets minimum recommended threshold, it falls below more conservative benchmarks [36]. The modest difference between R^2^ and adjusted R^2^ suggests limited overfitting, but the sample size warrants caution in interpreting the stability of individual predictor estimates, and replication in larger samples is recommended to confirm observed associations.

Fifth, social desirability may have influenced responses to attitudinal items, particularly those related to gender and skepticism about the legitimacy of INOCA. Prevalence of these attitudes may therefore reflect conservative estimates.

Finally, while organisational determinants were reported by individuals, direct assessment of institutional policies and practices was beyond the scope of this study.

## 5 Conclusion

Despite frequent clinical exposure to patients with (suspected) INOCA, familiarity with and uptake of the NVVC INOCA recommendation among Dutch cardiologists remains incomplete. Implementation currently appears to rely largely on the motivation of individual clinicians and their engagement, while organisational structures to support systematic uptake remain limited. As recognition of INOCA as a clinical condition grows within contemporary ischemic heart disease care, strengthening dissemination and embedding the recommendation within routine cardiology practice will be essential to translate emerging knowledge into more consistent diagnosis and management.

## Declaration of generative AI and AI-assisted technologies in the manuscript preparation process

During the preparation of this work the author(s) used LUMO (by Proton) in order to refine language and shorten sentence length. After using this tool/service, the author(s) reviewed and edited the content as needed and take(s) full responsibility for the content of the published article.

## CRediT authorship contribution statement

**Irene Göttgens:** Writing – review & editing, Writing – original draft, Supervision, Project administration, Methodology, Data curation, Conceptualization. **Lianne C.G. Roelofs:** Writing – review & editing, Project administration, Methodology, Investigation, Formal analysis, Data curation. **Jojanneke Smeenk:** Writing – review & editing, Methodology, Investigation. **Linda Modderkolk:** Writing – review & editing, Conceptualization. **Sabine Oertelt-Prigione:** Writing – review & editing, Supervision, Funding acquisition, Conceptualization.

## Availability of data and material

The datasets used and analyzed during the current study are available from the corresponding author upon reasonable request.

## Ethics approval and consent to participate

The study was approved by the institutional review board (IRB) of the Radboud University Medical Center (Dossier number: 2024-17221). All participant provided informed consent before participating in this study.

## Consent for publication

Not applicable.

## Declaration of competing interest

The authors declare no competing interests.

## References

[1] Odanovic N., Schwann A., Zhang Z. (2023). Long-term outcomes of patients with ischemia and no obstructive coronary artery disease (INOCA) - a systematic review and meta-analysis. Eur. Heart J..

[2] Tseng L.Y., Göç N., Schwann A.N. (2023). Illness perception and the impact of a definitive diagnosis on women with ischemia and no obstructive coronary artery disease: a qualitative study. Circ Cardiovasc Qual Outcomes.

[3] van Schalkwijk D.L., Widdershoven J., Magro M. (2023). Clinical and psychological characteristics of patients with ischemia and non-obstructive coronary arteries (INOCA) and obstructive coronary artery disease. Am. Heart J.: Cardiol. Res. Pract..

[4] Gulati M., Khan N., George M. (2023). Ischemia with no obstructive coronary artery disease (INOCA): a patient self-report quality of life survey from INOCA international. Int. J. Cardiol..

[5] Ranasinghe S., Merz C.N.B., Khan N. (2023). Sex differences in quality of life in patients with ischemia with no obstructive coronary artery disease (INOCA): a patient self-report retrospective survey from INOCA international. J. Clin. Med. Res..

[6] Burgess S., Cader F.A., Gulati M., Sutton N.R., Appelman Y., Banerjee S. (2025). Challenges in diagnosing coronary microvascular dysfunction and coronary vasospasm. Cardiovasc. Revascularization Med..

[7] Namba H.F., Boerhout C.K.M., Damman P. (2025). Invasive coronary function testing in clinical practice: implementing the 2024 ESC guidelines on chronic coronary syndromes. Int. J. Cardiol..

[8] NVVC Leidraad “Pijn Op De Borst Zonder Obstructief Coronairlijden”. Accessed September 11, 2023. https://www.nvvc.nl/Vereniging%20-%20bestuur/Nieuws/200629-geautoriseerd-nvvc-leidraad-pijn-op-de-borst-zonder-obstructief-coronairlijdennl.

[9] Fleuren M.A.H., Paulussen T.G.W.M., Van Dommelen P., Van Buuren S. (2014). Towards a measurement instrument for determinants of innovations. Int. J. Qual. Health Care.

[10] Francke A.L., Smit M.C., de Veer A.J.E., Mistiaen P. (2008). Factors influencing the implementation of clinical guidelines for health care professionals: a systematic meta-review. BMC Med. Inf. Decis. Making.

[11] Modderkolk L., Göttgens I., van den Hurk L., Oertelt-Prigione S. (2025). Barriers and facilitators to the uptake of the ischaemia with non-obstructive coronary arteries (INOCA) recommendation by cardiologists in the Netherlands: a qualitative study. Int J Cardiol Cardiovasc Risk Prev.

[12] Vorselaars V.M.M., Minneboo M., Meijers W.C. (2023). Career perspectives for young cardiologists in the Netherlands: a steady increase in temporary positions. Neth. Heart J..

[13] van Lummel E.V.T.J., Meijer Y., Tjan D.H.T., van Delden J.J.M. (2023). Barriers and facilitators for healthcare professionals to the implementation of multidisciplinary timely undertaken advance care planning conversations at the outpatient clinic (the MUTUAL intervention): a sequential exploratory mixed-methods study. BMC Palliat. Care.

[14] Remmits A.J.W., van Mastrigt G.A.P.G., Evers S.M.A.A., van Setten P.A. (2024). Facilitators and barriers to the transition from outpatient clinic visits to home-based check-ups for children being treated with growth hormone: a mixed-methods study. Eur. J. Pediatr..

[15] Hinteregger A., Niedermann K., Wirz M. (2023). The feasibility, facilitators, and barriers in the initial implementation phase of ‘good life with osteoarthritis in Denmark’ (GLA:D®) in Switzerland: a cross-sectional survey. BMC Health Serv. Res..

[16] Siebelink N.M., Gaasterland A., Gielissen M., van der Weegen S., Boon B., van der Poel A. (2024). Barriers and facilitators influencing implementation of care technology for people with intellectual disabilities: a cross-sectional study among care professionals. J. Appl. Res. Intellect. Disabil..

[17] Dugstad J., Sundling V., Nilsen E.R., Eide H. (2020). Nursing staff's evaluation of facilitators and barriers during implementation of wireless nurse call systems in residential care facilities. A cross-sectional study. BMC Health Serv. Res..

[18] Verberne L.M., Kars M.C., Schepers S.A., Schouten-van Meeteren A.Y.N., Grootenhuis M.A., van Delden J.J.M. (2018). Barriers and facilitators to the implementation of a paediatric palliative care team. BMC Palliat. Care.

[19] Habtezghi T., Haq A., Jin Y., Haq N., Bulluck H. (2025). Impact of coronary function testing on symptoms and quality of life in patients with coronary microvascular dysfunction: meta-analysis of randomised controlled trials. J. Clin. Med..

[20] McKee G., Kerins M., Hamilton G. (2017). Barriers to ESC guideline implementation: results of a survey from the european council on cardiovascular nursing and allied professions (CCNAP). Eur. J. Cardiovasc. Nurs..

[21] Agarwal A., Patel R., Khalique O.K. (2025). Coronary microvascular dysfunction: bridging the diagnosis-treatment divide in women with INOCA-A review. J. Clin. Med..

[22] Kunadian V., Raharjo D.E. (2022). Breaking down the barriers in the management of INOCA: how can we do better in the diagnosis of coronary vasomotor disorders?. EuroIntervention.

[23] Crooijmans C., Jansen T.P.J., Konst R.E. (2023). Design and rationale of the NetherLands registry of invasive coronary vasomotor function testing (NL-CFT). Int. J. Cardiol..

[24] Wang T., Tan J.Y.B., Liu X.L., Zhao I. (2023). Barriers and enablers to implementing clinical practice guidelines in primary care: an overview of systematic reviews. BMJ Open.

[25] Hoorn C.J.G.M., Crijns H.J.G.M., Dierick-van Daele A.T.M., Dekker L.R.C. (2019). Review on factors influencing physician guideline adherence in cardiology. Cardiol. Rev..

[26] Di Nuovo F., Onorati M., Nicola M., Cassoni M. (2018). Looking into the differences of physician gender in medical practice. Journal of Sex- and Gender-Specific Medicine.

[27] Temkin S.M., Salles A., Barr E., Leggett C.B., Reznick J.S., Wong M.S. (2024). ‘Women's work’: gender and the physician workforce. Soc. Sci. Med..

[28] Kunadian V., Chieffo A., Camici P.G. (2021). An EAPCI expert consensus document on ischaemia with non-obstructive coronary arteries in collaboration with european society of cardiology working group on coronary pathophysiology & microcirculation endorsed by coronary vasomotor disorders international study group. EuroIntervention.

[29] Vrints C., Andreotti F., Koskinas K.C. (2024). 2024 ESC guidelines for the management of chronic coronary syndromes. Eur. Heart J..

[30] Gender W. (2020). NVVC Leidraad Pijn Op de Borst Zonder Obstructief Coronairlijden. Nederlandse Vereniging voor Cardiologie.

[31] Pereira V.C., Silva S.N., Carvalho V.K.S., Zanghelini F., Barreto J.O.M. (2022). Strategies for the implementation of clinical practice guidelines in public health: an overview of systematic reviews. Health Res. Pol. Syst..

[32] Cochrane. Audit and feedback: effects on professional practice. Published online 25 March 2025. Accessed February 9, 2026. https://www.cochrane.org/evidence/CD000259_audit-and-feedback-effects-professional-practice.

[33] Powell B.J., Waltz T.J., Chinman M.J. (2015). A refined compilation of implementation strategies: results from the expert recommendations for implementing change (ERIC) project. Implement. Sci..

[34] Mitton C., Adair C.E., McKenzie E., Patten S.B., Waye Perry B. (2007). Knowledge transfer and exchange: review and synthesis of the literature: knowledge transfer and exchange. Milbank Q..

[35] Van Schalkwijk D.L., Widdershoven J.W.M.G., Elias-Smale S. (2023). ShareHeart: a patient journey map of patients with ischemia and non-obstructive coronary artery disease based on qualitative research. J. Clin. Nurs..

[36] Seabrook J.A. (2025). Powering nutrition research: practical strategies for sample size in multiple regression. Nutrients.

