# Supplementary material for: Barriers and facilitators for the uptake of the national INOCA recommendation among Dutch cardiologists: A survey study
Source: Int J Cardiol Cardiovasc Risk Prev. 2026 Oct 8;31:200718. doi: 10.1016/j.ijcrp.2026.200718 (PMC13681466; doi:10.1016/j.ijcrp.2026.200718)
Supplement: Supplementary file 1 [file mmc1.docx]

# Supplementary materials

Paper title: “*Barriers and facilitators to the uptake of Ischaemia with Non-Obstructive Coronary Arteries (INOCA) Recommendations by cardiologist in the Netherlands: A national survey study”*

Authors: Irene Göttgens, Lianne Roelofs, Jojanneke Smeenk, Lori van den Hurk, Linda Modderkolk, Sabine Oertelt-Prigione

Table of Contents

[Supplementary materials	1](#_Toc237278963)

[Appendix A. Recruitment Strategy	2](#_Toc237278964)

[Appendix B. Respondent Flow (CONSORT-style diagram)	4](#_Toc237278965)

[Appendix C. ODI-INOCA Survey Questions	4](#_Toc237278966)

[Appendix D. Determinants for implementation of the NVVC INOCA Recommendation – complete overview MIDI results	16](#_Toc237278967)

### Appendix A. Recruitment Strategy

**Context and Rationale**

Cardiologists are a hard-to-reach professional group for survey research due to demanding clinical schedules and limited availability. To maximize reach and representativeness, we employed a multimodal recruitment strategy. Based on the available data, approximately 1100 cardiologists were eligible to receive the invitation.^1^ We aimed for an inclusion rate of approximately 10% (N=110 inclusions).

**Strategy design**

A targeted communication strategy was developed to recruit participating cardiologists. The aim was to effectively inform them about the study and to motivate participation in the questionnaire. The communication was structured into three phases, which could be individually adjusted during the process based on ongoing insights:

(1) preparation of materials;

(2) active recruitment through events and digital channels;

(3) follow-up and completion.

This structured approach ensured consistency and effectiveness in recruitment efforts.

To operationalize the strategy, the recruitment team addressed the following key points:

- Which information is most relevant to share, focusing on the scientific background and the potential clinical relevance for cardiology practice;
- Which tone of voice needs to be used, especially for cardiologists;
- What is the most effective setting to approach cardiologists;
- When is the best time for recruitment.

**Communication materials**

The following communication materials were developed:

- Presentation slide;
- Participant information letter;
- Digital flyer;
- Recruitment messages (initial and follow up) for institutional organizations to share directly with their cardiologists;
- Invitation messages (initial and follow up) to be sent to institutional organizations with the aim of encouraging them to disseminate the message through their professional networks.
- Social media invitations (for dissemination via professional Linkedin networks)

The content and tone of the message were tailored to what resonates with cardiologists: contributing to improved patient care and advancing knowledge within the field. The messages were formulated with consideration of the heavy clinical workload of cardiologists, ensuring that the communication remained concise, focused, and actionable. Since the perspectives on the study topic (INOCA) vary among cardiologists, the recruitment materials were designed in a factual, credible, and empathetic tone, avoiding any one-sided framing. For this reason, as well, the strategy emphasized personal communication, as direct contact—through individual invitations or in-person discussions at professional events—was considered more effective in fostering goodwill and personal engagement than (only) general calls within networks.

**Active recruitment**

A communication plan was developed based on the available timeline from April to September. In developing the plan, periods when cardiologists are most accessible—such as around relevant professional events—as well as their reduced availability during holiday periods were taken into account.

A variety of digital formats and channels were used in a complementary manner to attract attention in different ways. The dissemination was organized in successive periods to avoid overwhelming recipients with INOCA content in a short period of time, to capitalize on the effectiveness of repetition and to maintain study visibility over time.

**Channels**

The target group was reached through both physical and digital channels: personal contact during conferences with representatives of professional networks and meetings, complemented by digital communication via professional networks and direct e-mail invitations. This combination enabled both broad visibility and individual engagement.

- The following **institutional organizations** were invited by email to disseminate the survey link via **direct mailings**, **departmental newsletters** and **intranet announcements**:
  - Cardiology Associations
  - Cardiology Centers
  - University Medical Centers & Independent Cardiovascular Centers
  - Peripheral Hospitals
  - Patient Advocacy Initiatives
  - Members of the original NVVC INOCA Guideline committee
- **Professional networks**: members of the study team circulated invitations through personal and professional networks.
- **Conferences and meetings**: the study was highlighted at the NVVC Congress and the IMPRESS Consortium Meeting.
- **LinkedIn:** The principle investigators shared invitations to complete the survey. Additionally, cardiologists were personally invited via LinkedIn to complete the study.

**Invitation and Reminders**

The survey was open between April–September, 2025. The initial invitation to the institutional organizations was followed by one reminder email after a one-week interval only if no response was received. This was followed by three or four follow ups sent at eight-week intervals, including a follow-up phone call if still no follow-up response was received. Each invitation emphasized the voluntary and anonymous nature of participation and the estimated time commitment (5–10 minutes for the full survey, 2–3 minutes for the short version). In total 163 invitations were sent to institutional and organizational representatives for distribution among their professional networks. Through these initial invitations, a total of 177 cardiologists agreed to participate in the study (provided informed consent), of which 134 cardiologists completed the minimal dataset for inclusion (Appendix B). Reasons for dropout were not recorded.

**Eligibility and Consent**

All practicing cardiologists in the Netherlands were eligible. Interested participants accessed the survey via a secure, GDPR-compliant platform. After reading the study information, participants provided digital informed consent before proceeding.

**Response Rate**
In total, 147 respondents accessed the survey, of whom 134 completed it. The inclusion rate was therefore estimated at 12%. 78 Respondents submitted the full questionnaire and 56 the shortened version.

### Appendix B. Respondent Flow (CONSORT-style diagram)

### Appendix C. ODI-INOCA Survey Questions

**Demografische gegevens**

Hartelijk dank voor uw deelname aan dit onderzoek. Allereerst zouden we u graag een aantal algemene vragen stellen over uw achtergrond.

1. Wat is uw genderidentiteit?

- Vrouw
- Man
- Non-binair
- Zeg ik liever niet
- Anders, namelijk:

2. Hoeveel jaar bent u werkzaam als cardioloog?

- Minder dan 1 jaar
- 1 tot 5 jaar
- 6 tot 15 jaar
- Meer dan 15 jaar

3. In welk type zorg instelling bent u momenteel werkzaam?

- Een perifeer ziekenhuis
- Een academisch ziekenhuis
- Een Cardiologie Centra Nederland (CCN)
- Anders, namelijk:

In 2020 heeft de Nederlandse Vereniging Voor Cardiologie (NVVC) een [leidraad](https://www.nvvc.nl/Richtlijnen/NVVC%20Leidraad%20AP%20zonder%20obstructief%20conorairlijden%20def%20versie_24-6-20.pdf) voor cardiologen opgesteld die gericht is op het adequaat herkennen, diagnosticeren en behandelen van patiënten met een (verdenking op) INOCA (Ischemia and No Obstructive Coronary Artery Disease). Klik [hier](https://www.nvvc.nl/Richtlijnen/NVVC%20Leidraad%20AP%20zonder%20obstructief%20conorairlijden%20def%20versie_24-6-20.pdf) indien u de one-pager van de leidraad wilt bekijken.

1. Bent u bekend met de NVVC INOCA leidraad?
   - Ja
   - Nee [door naar vraag 3]
2. In hoeverre bent u vertrouwd met de inhoud van de NVVC INOCA leidraad en de specifieke aanbevelingen daarin?
   - Ik ken de leidraad en de aanbevelingen volledig uit mijn hoofd en kan deze zonder raadpleging toepassen.
   - Ik ben goed bekend met de leidraad en de meeste aanbevelingen, maar voor sommige specifieke details moet ik de documentatie raadplegen.
   - Ik ken de grote lijnen van de leidraad, maar moet de specifieke aanbevelingen opzoeken om ze correct toe te passen.
   - Ik ben enigszins vertrouwd met de leidraad, maar ik moet deze regelmatig raadplegen voor zowel de algemene als specifieke aanbevelingen.
   - Ik ben niet goed vertrouwd met de leidraad en moet deze altijd raadplegen bij het toepassen van de aanbevelingen.
3. Ziet u in uw werk patiënten met een (verdenking van) INOCA diagnose?
   - Ja [door naar vraag 4]
   - Nee [door naar vraag 5 of indien ‘nee’ bij vraag 1 naar einde vragenlijst]
4. Hoeveel patiënten met (verdenking van) een INOCA diagnose ziet u naar schatting per maand?

**Algemene stellingen INOCA**

Er volgen nu een aantal algemene stellingen met betrekking tot INOCA. Geef alstublieft voor iedere stelling aan in welke mate u het hier mee eens bent.

5. Ik merk dat de kennis van het diagnose- en behandelproces voor INOCA-patiënten de afgelopen jaren is verbeterd.
   - Helemaal niet mee eens
   - Niet mee eens
   - Neutraal
   - Mee eens
   - Helemaal mee eens
6. Ik denk dat er niet genoeg bekend is over INOCA, en dat maakt me sceptisch over of het een legitieme aandoening is.
   - Helemaal niet mee eens
   - Niet mee eens
   - Neutraal
   - Mee eens
   - Helemaal mee eens
7. Ik vind het belangrijk om de symptomen en klachten van patiënten met (verdenking van) INOCA te erkennen en serieus te nemen, zelfs als er klinische onzekerheid is over hun aandoening.
   - Helemaal niet mee eens
   - Niet mee eens
   - Neutraal
   - Mee eens
   - Helemaal mee eens
8. Er zijn onvoldoende goede diagnostische middelen en behandelopties beschikbaar voor INOCA-patiënten.
   - Helemaal niet mee eens
   - Niet mee eens
   - Neutraal
   - Mee eens
   - Helemaal mee eens
9. Ik ben in staat om effectief te communiceren met patiënten met (een verdenking van) INOCA, zelfs als er klinische onzekerheid is over hun aandoening**.**
   - Helemaal niet mee eens
   - Niet mee eens
   - Neutraal
   - Mee eens
   - Helemaal mee eens
10. Het beeld dat INOCA-patiënten vooral vrouwen zijn, vind ik een stereotypering.
    - Helemaal niet mee eens
    - Niet mee eens
    - Neutraal
    - Mee eens
    - Helemaal mee eens
11. Ik voel me gesteund door collega’s en experts in de zorg die ik verleen aan patiënten met (verdenking van) INOCA.
    - Helemaal niet mee eens
    - Niet mee eens
    - Neutraal
    - Mee eens
    - Helemaal mee eens
12. Ik ervaar de zorg voor patiënten met (een verdenking van) INOCA als bijzonder veeleisend en gecompliceerd.
    - Helemaal niet mee eens
    - Niet mee eens
    - Neutraal
    - Mee eens
    - Helemaal mee eens

**2020 NVVC INOCA leidraad**

Er volgen nu een aantal stellingen die gaan over de leidraad. Mocht u, voordat u hier mee verder gaat, de samenvatting van de leidraad nogmaals willen bekijken, klik dan [hier](https://www.nvvc.nl/Richtlijnen/NVVC%20Leidraad%20AP%20zonder%20obstructief%20conorairlijden%20def%20versie_24-6-20.pdf).

Geef alstublieft voor iedere stelling aan in welke mate u het hier mee eens bent.

13. De leidraad is relevant voor mij in mijn werk.
    - Helemaal mee oneens
    - Mee oneens
    - Noch mee oneens, noch mee eens
    - Mee eens
    - Helemaal mee eens

Wilt u toelichten waarom u deze leidraad wel/niet relevant vindt in uw werk?

14. De leidraad geeft helder aan welke activiteiten ik in welke volgorde moet uitvoeren.
    - Helemaal mee oneens
    - Mee oneens
    - Noch mee oneens, noch mee eens
    - Mee eens
    - Helemaal mee eens
15. De leidraad is gebaseerd op feitelijk juiste kennis.
    - Helemaal mee oneens
    - Mee oneens
    - Noch mee oneens, noch mee eens
    - Mee eens
    - Helemaal mee eens
16. De leidraad biedt alle informatie en materialen die nodig zijn om er goed mee te kunnen werken.
    - Helemaal mee oneens
    - Mee oneens
    - Noch mee oneens, noch mee eens
    - Mee eens
    - Helemaal mee eens
17. De leidraad is te ingewikkeld voor mij om te kunnen toepassen.
    - Helemaal mee oneens
    - Mee oneens
    - Noch mee oneens, noch mee eens
    - Mee eens
    - Helemaal mee eens
18. De leidraad sluit goed aan bij hoe ik gewend ben om te werken.
    - Helemaal mee oneens
    - Mee oneens
    - Noch mee oneens, noch mee eens
    - Mee eens
    - Helemaal mee eens
19. Ik vind de effecten van het gebruik van de leidraad duidelijk zichtbaar.
    - Helemaal mee oneens
    - Mee oneens
    - Noch mee oneens, noch mee eens
    - Mee eens
    - Helemaal mee eens
20. Ik vind de leidraad geschikt voor mijn patiënten met (verdenking van) INOCA.
    - Helemaal mee oneens
    - Mee oneens
    - Noch mee oneens, noch mee eens
    - Mee eens
    - Helemaal mee eens
21. Het volgen van de leidraad biedt voor mij persoonlijk voordelen.
    - Helemaal mee oneens
    - Mee oneens
    - Noch mee oneens, noch mee eens
    - Mee eens
    - Helemaal mee eens
22. (Indien antwoord ‘Mee eens’ of ‘Helemaal mee eens’ ) Wilt u toelichten welke voordelen dit zijn?
23. Het volgen van de leidraad biedt voor mij persoonlijk nadelen.
    - Helemaal mee oneens
    - Mee oneens
    - Noch mee oneens, noch mee eens
    - Mee eens
    - Helemaal mee eens
24. (Indien antwoord ‘Mee eens’ of ‘Helemaal mee eens’ ) Wilt u toelichten welke nadelen dit zijn?
25. Ik *vind het belangrijk* om met de leidraad de volgende doelstellingen te bereiken:

a. Dat cardiologen patiënten met (verdenking op) INOCA adequaat kunnen **herkennen**.

- Helemaal mee oneens
- Mee oneens
- Noch mee oneens, noch mee eens
- Mee eens
- Helemaal mee eens

b. Dat cardiologen patiënten met (verdenking op) INOCA adequaat kunnen **diagnosticeren**.

- Helemaal mee oneens
- Mee oneens
- Noch mee oneens, noch mee eens
- Mee eens
- Helemaal mee eens

c. Dat cardiologen patiënten met (verdenking op) INOCA adequaat kunnen **behandelen**.

- Helemaal mee oneens
- Mee oneens
- Noch mee oneens, noch mee eens
- Mee eens
- Helemaal mee eens

26. Ik *verwacht* dat met de leidraad de volgende doelstellingen worden bereikt:

a. Dat cardiologen patiënten met (verdenking op) INOCA adequaat kunnen **herkennen**.

- Zeer zeker niet
- Zeker niet
- Misschien niet, misschien wel
- Zeker wel
- Zeer zeker wel

b. Dat cardiologen patiënten met (verdenking op) INOCA adequaat kunnen **diagnosticeren**.

- Zeer zeker niet
- Zeker niet
- Misschien niet, misschien wel
- Zeker wel
- Zeer zeker wel

c. Dat cardiologen patiënten met (verdenking op) INOCA adequaat kunnen **behandelen**.

- Zeer zeker niet
- Zeker niet
- Misschien niet, misschien wel
- Zeker wel
- Zeer zeker wel

27. Ik vind het tot mijn functie horen om deze leidraad te volgen.
    - Helemaal mee oneens
    - Mee oneens
    - Noch mee oneens, noch mee eens
    - Mee eens
    - Helemaal mee eens
28. Patiënten zullen over het algemeen tevreden zijn als ik deze leidraad volg.
    - Helemaal mee oneens
    - Mee oneens
    - Noch mee oneens, noch mee eens
    - Mee eens
    - Helemaal mee eens
29. Patiënten zullen over het algemeen mijn aanbevelingen volgen als ik deze leidraad volg.
    - Helemaal mee oneens
    - Mee oneens
    - Noch mee oneens, noch mee eens
    - Mee eens
    - Helemaal mee eens
30. Ik kan op voldoende hulp van mijn collega’s rekenen mocht ik die nodig hebben bij het volgen van de leidraad.
    - Helemaal mee oneens
    - Mee oneens
    - Noch mee oneens, noch mee eens
    - Mee eens
    - Helemaal mee eens
31. Ik kan op voldoende hulp van mijn leidinggevende rekenen mocht ik die nodig hebben bij het volgen van de leidraad.
    - Helemaal mee oneens
    - Mee oneens
    - Noch mee oneens, noch mee eens
    - Mee eens
    - Helemaal mee eens
32. Ik kan op voldoende hulp van betrokken professionals in de zorgketen rekenen mocht ik die nodig hebben bij het volgen van de leidraad.
    - Helemaal mee oneens
    - Mee oneens
    - Noch mee oneens, noch mee eens
    - Mee eens
    - Helemaal mee eens
33. Hoe groot is volgens u het deel collega’s in uw organisatie voor wie de leidraad is bedoeld, die de leidraad ook daadwerkelijk gebruiken?
    - Geen enkele collega
    - Bijna geen enkele collega
    - Een minderheid
    - De helft
    - Een meerderheid
    - Bijna alle collega’s
    - Alle collega’s
34. In hoeverre verwachten de volgende personen dat u de leidraad volgt:

a. patiënten

- Zeer zeker niet
- Zeker niet
- Misschien niet, misschien wel
- Zeker wel
- Zeer zeker wel

b. uw collega-cardiologen

- Zeer zeker niet
- Zeker niet
- Misschien niet, misschien wel
- Zeker wel
- Zeer zeker wel

c. uw direct leidinggevende

- Zeer zeker niet
- Zeker niet
- Misschien niet, misschien wel
- Zeker wel
- Zeer zeker wel

d. andere betrokken professionals in de zorgketen

- Zeer zeker niet
- Zeker niet
- Misschien niet, misschien wel
- Zeker wel
- Zeer zeker wel

35. (Indien 34d ‘Zeker wel’ of ‘Zeer zeker wel’) U heeft aangegeven dat andere betrokken professionals in de zorgketen van u verwachten dat u de leidraad volgt. Wie zijn dit?
36. Als het gaat om het volgen van de leidraad, hoeveel trekt u zich dan aan van de mening van de volgende personen:

a. patiënten

- Zeer weinig
- Weinig
- Niet weinig, niet veel
- Veel
- Zeer veel

b. uw collega-cardiologen

- Zeer weinig
- Weinig
- Niet weinig, niet veel
- Veel
- Zeer veel

c. uw direct leidinggevende

- Zeer weinig
- Weinig
- Niet weinig, niet veel
- Veel
- Zeer veel

d. andere betrokken professionals in de zorgketen

- Zeer weinig
- Weinig
- Niet weinig, niet veel
- Veel
- Zeer veel

37. (Indien 36d ‘Zeker wel’ of ‘Zeer zeker wel’) U heeft aangegeven zich veel of zeer veel aan te trekken van de mening van andere betrokken professionals in de zorgketen als het gaat om het volgen van de leidraad. Wie zijn dit?
38. Indien u dat zou willen, denkt u dat het u dan lukt om alle stappen van de leidraad op te volgen bij alle patiënten met verdenking op INOCA die u in zorg heeft?
    - Zeer zeker niet
    - Zeker niet
    - Misschien niet, misschien wel
    - Zeker wel
    - Zeer zeker wel
39. Ik beschik over voldoende kennis om de leidraad te kunnen volgen.
    - Helemaal mee oneens
    - Mee oneens
    - Noch mee oneens, noch mee eens
    - Mee eens
    - Helemaal mee eens
40. In hoeverre bent u op de hoogte van de inhoud van de leidraad?
    - Ik ken de leidraad niet
    - Ik ken de leidraad wel, maar heb hem (nog) niet doorgelezen
    - Ik ken de leidraad en heb hem oppervlakkig doorgelezen
    - Ik ken de leidraad en heb hem volledig en grondig gelezen
41. Zijn in uw organisatie formeel afspraken vastgelegd door het management over het gebruik van de leidraad (in beleidsplannen, werkplannen en dergelijke)?
    - Nee
    - Ja
42. In mijn organisatie zijn maatregelen getroffen zodat medewerkers die de leidraad gebruiken en de organisatie verlaten, tijdig worden vervangen door (nieuwe) medewerkers die voldoende zijn/worden ingewerkt in de leidraad.
    - Helemaal mee oneens
    - Mee oneens
    - Noch mee oneens, noch mee eens
    - Mee eens
    - Helemaal mee eens
43. Er is voldoende personeel in onze organisatie om de leidraad zoals bedoeld te kunnen volgen.
    - Helemaal mee oneens
    - Mee oneens
    - Noch mee oneens, noch mee eens
    - Mee eens
    - Helemaal mee eens
44. Er zijn voldoende financiële middelen beschikbaar om de leidraad zoals bedoeld te kunnen volgen.
    - Helemaal mee oneens
    - Mee oneens
    - Noch mee oneens, noch mee eens
    - Mee eens
    - Helemaal mee eens
45. Onze organisatie stelt mij voldoende tijd beschikbaar om de leidraad zoals bedoeld te integreren in mijn dagelijks werk.
    - Helemaal mee oneens
    - Mee oneens
    - Noch mee oneens, noch mee eens
    - Mee eens
    - Helemaal mee eens
46. Onze organisatie stelt mij voldoende materialen en voorzieningen beschikbaar om de leidraad zoals bedoeld te kunnen volgen.
    - Helemaal mee oneens
    - Mee oneens
    - Noch mee oneens, noch mee eens
    - Mee eens
    - Helemaal mee eens
47. (Indien 46 ‘Helemaal mee oneens’ of ‘Mee oneens’) U heeft aangegeven dat bepaalde materialen en voorzieningen onvoldoende beschikbaar zijn in uw organisatie om de leidraad zoals bedoeld te kunnen volgen. Om welke materialen gaat dit?
48. In mijn organisatie is/zijn één of meerdere personen aangewezen voor het coördineren van de invoering van de leidraad.
    - Nee
    - Ja
49. Zijn er, behalve de invoering van de leidraad, andere veranderingen waarmee u momenteel of binnen afzienbare tijd mee te maken heeft?
    - Nee
    - Ja
50. Ik heb in mijn organisatie makkelijk toegang tot informatie over het gebruik van de leidraad.
    - Helemaal mee oneens
    - Mee oneens
    - Noch mee oneens, noch mee eens
    - Mee eens
    - Helemaal mee eens
51. In mijn organisatie vindt regelmatig terugkoppeling plaats over de voortgang van de invoering van de leidraad.
    - Helemaal mee oneens
    - Mee oneens
    - Noch mee oneens, noch mee eens
    - Mee eens
    - Helemaal mee eens
52. De activiteiten die in de leidraad staan, sluiten goed aan bij bestaande wetten en regels.
    - Helemaal mee oneens
    - Mee oneens
    - Noch mee oneens, noch mee eens
    - Mee eens
    - Helemaal mee eens
53. Zijn er nog dingen die u belangrijk vindt om ons mee te geven met betrekking tot de implementatie van de INOCA leidraad dat nog niet aan bod is gekomen in deze vragenlijst?
54. Zou u er voor openstaan om een uitnodiging te ontvangen voor deelname aan een vervolgonderzoek vanuit de IMPRESS onderzoeksgroep met betrekking tot het verbeteren van zorg voor patiënten met INOCA?
    - Ja
    - Nee
55. (Indien 54 ‘ja’) Op welk emailadres mogen we u hiervoor benaderen?

### Appendix D. Determinants for implementation of the NVVC INOCA Recommendation – complete overview MIDI results

|  | **Determinants** | **Item** | **Positive response (%)**  **≥80% (bold) = facilitator** | | | **Negative response (%) ≥20% (bold) = barrier** | | |
| --- | --- | --- | --- | --- | --- | --- | --- | --- |
|  |  |  | **Women** | **Men** | **Total** | **Women** | **Men** | **Total** |
| **Innovation** | |  |  |  |  |  |  |  |
| D1 | Procedural clarity | The guideline clearly describes the activities I should perform and in which order. | 79,4 | 72,7 | 75,6 | 8,8 | 4,5 | 6,4 |
| D2 | Correctness | The guideline is based on factually correct knowledge. | 79,4 | 75,0 | 76,9 | 0 | 2,3 | 1,3 |
| D3 | Completeness | The guideline provides all the information and materials needed to work with it properly. | 73,5 | 54,5 | 62,8 | 14,7 | 11,4 | 12,8 |
| D4 | Complexity | The guideline is easy for me to use. | **100** | 77,3 | **87,2** | 0 | 11,4 | 6,4 |
| D5 | Compatibility | The guideline is a good match for how I am used to working. | 79,4 | 72,7 | 75,6 | 0 | 2,3 | 1,3 |
| D6 | Observability | The outcomes of using the guideline are clearly observable. | 44,1 | 45,5 | 44,9 | **23,5** | 11,4 | 16,7 |
| D7 | Relevance for client | I think the guideline is relevant for my patients with (suspected) INOCA. | **85,3** | 72,7 | 78,2 | 2,9 | 6,8 | 5,1 |
| **User** | |  |  |  |  |  |  |  |
| D8a | Personal benefits | Following the guideline has personal benefits for me. | 44,1 | 40,9 | 42,3 | 11,8 | 11,4 | 11,5 |
| D8b | Personal drawbacks | Following the guideline has personal drawbacks for me. | 55,9 | 61,4 | 59,0 | 8,8 | 4,5 | 6,4 |
| D9 | Outcome expectations | **I believe it is important to achieve the following objectives with the guideline:** |  |  |  |  |  |  |
|  |  | *Cardiologists can adequately* ***recognise*** *patients with (suspected) INOCA* | **97,1** | **100** | **98,7** | 2,9 | 0 | 1,3 |
|  |  | *Cardiologists can adequately* ***diagnose*** *patients with (suspected) INOCA* | **91,2** | **88,6** | **89,7** | 2,9 | 0 | 1,3 |
|  |  | *Cardiologists can adequately* ***treat*** *patients with (suspected) INOCA* | **100** | **97,7** | **98,7** | 0 | 0 | 0 |
|  |  | **I expect the guidelines to achieve the following objectives:** |  |  |  |  |  |  |
|  |  | *Cardiologists can adequately* ***recognise*** *patients with (suspected) INOCA* | 76,5 | 75,0 | 75,6 | 0 | 0 | 0 |
|  |  | *Cardiologists can adequately* ***diagnose*** *patients with (suspected) INOCA* | 67,6 | 77,3 | 73,1 | 5,9 | 2,3 | 3,8 |
|  |  | *Cardiologists can adequately* ***treat*** *patients with (suspected) INOCA* | 76,5 | 79,5 | 78,2 | 5,9 | 0 | 2,6 |
|  |  |  |  |  |  |  |  |  |
| D10 | Professional obligation | I feel it is my responsibility as a professional to use this guideline. | **91,2** | **88,6** | **89,7** | 0 | 2,3 | 1,3 |
| D11 | Client satisfaction | Patients will generally be satisfied if I use this guideline. | 67,6 | 52,3 | 59,0 | 5,9 | 4,5 | 5,1 |
| D12 | Client cooperation | Patients will generally follow my recommendations when I use the guideline. | 64,7 | 54,5 | 59,0 | 2,9 | 4,5 | 3,8 |
| D13a | Social support | I can count on adequate assistance from *my colleagues* if I need it to use the guideline. | 79,4 | 70,5 | 74,4 | 5,9 | 6,8 | 6,4 |
| D13b |  | I can count on adequate assistance from *my supervisor* if I need it to use the guideline. | 50,0 | 47,7 | 48,7 | 5,9 | 6,8 | 6,4 |
| D13c |  | I can count on adequate assistance from *relevant professionals in the care chain* if I need it to use the guideline. | 76,5 | 72,7 | 74,4 | 8,8 | 0 | 3,8 |
| D14 | Descriptive norm | In your opinion, what proportion of the colleagues in your organisation for whom the guideline is intended, actually use the guideline? | 41,2 | 47,7 | 44,9 | **38,2** | **31,8** | **34,6** |
| D15a | Subjective norm – Normative beliefs | To what extent do the following people expect you to use the  guideline? |  |  |  |  |  |  |
|  |  | *Patients* | 67,6 | 59,1 | 62,8 | 11,8 | 9,1 | 10,3 |
|  |  | *Your fellow cardiologists* | 58,8 | 61,4 | 60,3 | 5,9 | 2,3 | 3,8 |
|  |  | *Your immediate supervisor* | 47,1 | 45,5 | 46,2 | 11,8 | 11,4 | 11,5 |
|  |  | *Other professionals involved in the care chain* | 44,1 | 34,1 | 38,5 | 5,9 | 9,1 | 7,7 |
| D15b | Subjective norm –Motivation to comply | When it comes to working in accordance with the guideline, to what extent do you comply with the opinions of the following people? |  |  |  |  |  |  |
|  |  | *Patients* | 67,6 | 68,2 | 67,9 | 5,9 | 4,5 | 5,1 |
|  |  | *Your fellow cardiologists* | 55,9 | 65,9 | 61,5 | 14,7 | 9,1 | 11,5 |
|  |  | *Your immediate supervisor* | 23,5 | 25,0 | 24,4 | **20,6** | 13,6 | 16,7 |
|  |  | *Other professionals involved in the care chain* | 20,6 | 29,5 | 25,6 | **20,6** | 6,8 | 12,8 |
| D16 | Self-efficacy | Should you wish to do so, do you think you would be able to follow all the steps in the guideline into practice? | 47,1 | 34,1 | 39,7 | **20,6** | 18,2 | 19,2 |
| D17 | Knowledge | I know enough to use the guideline. | **94,1** | **88,6** | **91,0** | 0 | 2,3 | 1,3 |
| **Organisation** | |  |  |  |  |  |  |  |
| D19 | Formal ratification by management | Has the management set up formal arrangements in your organisation relating to the use of this guideline (in policy plans, work plans and so on)? ** | 26,5 | 25,0 | 25,6 | **73,5** | **75,0** | **74,4** |
| D20 | Replacement when staff leave | In my organisation, there are arrangements in place so that staff who use the guideline and leave the organisation are replaced in good time by employees who are/will be adequately prepared to take over. | 14,7 | 29,5 | 23,1 | **38,2** | **45,5** | **42,3** |
| D21 | Staff capacity | There are enough people in our organisation to use the guideline as intended. | 70,6 | 54,5 | 61,5 | 11,8 | **22,7** | 17,9 |
| D22 | Financial resources | There are enough financial resources available to use the guideline as intended. | 58,8 | 59,1 | 59,0 | 11,8 | 15,9 | 14,1 |
| D23 | Time available | Our organisation provides me with enough time to include the guideline as intended in my day-to-day work. | 50,0 | 56,8 | 53,8 | **26,5** | 18,2 | **21,8** |
| D24 | Material resources and facilities | Our organisation provides me with enough materials and other resources or facilities necessary for the use of the guideline as intended. | 55,9 | 54,5 | 55,1 | 14,7 | 9,1 | 11,5 |
| D25 | Coordinator | In my organisation, one or more people have been designated to coordinate the process of implementing guideline. ** | 29,4 | 38,6 | 34,6 | **70,6** | **61,4** | **65,4** |
| D26 | Unsettled organisation | Are there, in addition to the implementation of the guideline, any other changes in the organisation affecting the implementation of the innovation now or in the foreseeable future (reorganisation, merger, cuts, staffing changes, other innovations)? ** | 79,4 | 63,6 | 70,5 | **20,6** | **36,4** | **29,5** |
| D27 | Information accessible about use of innovation | It is easy for me to find information in my organisation about using the guideline as intended. | 79,4 | 70,5 | 74,4 | 2,9 | 11,4 | 7,7 |
| D28 | Performance feedback | In my organisation, feedback is regularly provided about progress with the implementation of the guideline. | 11,8 | 13,6 | 12,8 | **64,7** | **52,3** | **57,7** |
| **Socio-political context** | |  |  |  |  |  |  |  |
| D29 | Legislation and regulations | The activities listed in the guideline fit in well with existing legislation and regulations. | 64,7 | 40,9 | 51,3 | 2,9 | 4,5 | 3,8 |
